# Assessment of psychometric properties of the questionnaire on supervisor-doctoral student interaction (QSDI) in Iran

**DOI:** 10.1186/s12909-021-02932-0

**Published:** 2021-10-14

**Authors:** Hooman Daryoushi, Amir Jalali, Ehsan Karimi, Nader Salari, Parvin Abbasi

**Affiliations:** 1 Department of Pediatrics, School of Medicine, Kermanshah University of Medical Sciences, Kermanshah, Iran; 2 Substance Abuse Prevention Research Center, Research Institute for Health, Kermanshah University of Medical Sciences, Kermanshah, Iran; 3 Student Research Committee, School of Nursing and Midwifery, Kermanshah University of Medical Sciences, Kermanshah, Iran; 4 Department of Biostatistics, School of Public Health, Kermanshah University of Medical Sciences, Kermanshah, Iran; 5 Department of Nursing, School of Nursing and Midwifery, Kermanshah University of Medical Sciences, Kermanshah, Iran

**Keywords:** Validity, Reliability, Student-supervisor interaction, Persian

## Abstract

**Background:**

One of the main elements that help students in research projects and composing dissertations is the student-supervisor relationship. A valid and reliable tool to measure this seems essential and it is the objective of the present study to validate and assess the psychometric properties of a questionnaire on supervisor-doctoral student interaction (QSDI) in Iran.

**Methods:**

Before starting the study, a permission from the developer of the tool was secured. Then the tool was forward-backward translated. After preparing the Farsi version of the tool, content validity was confirmed through qualitative and quantitative methods. To examine construct validity, exploratory factor analysis (EFA) and confirmatory factor analysis (CFA) were conducted with participation of 218 and 410 MD, MSc, and PhD students of medical sciences, respectively. To check reliability of the tool, correlation coefficient was used. To examine internal consistency of the tool, Cronbach’s alpha was used. Data analyses were done in SPSS (v.25) and LISREL (v.8).

**Results:**

The EFA and CFA results revealed eight factors and 39 items. The value of R-square for the model was equal to 0.99, which means 99% of changes in the dependent variable (supervisor-student interaction) is attributed to the independent variable (41 items). That is, 99% of the dependent variable changes is due to the independent variables. The main indices of the model based on factor analyses were supported (0.9<), which indicated goodness of fit of the model (χ2/df = 1.76, CFI, NFI, TLI = 0.98 GFI = 0.91, RMSEA = 0.043, R-square = 0.99). The significance level for correlation coefficient was below 0.05. Reliability of the tool was supported based on internal correlation (Cronbach’s alpha) equal to 0.943 for the whole tool and in 0.89–0.97 range for the subscales.

**Conclusion:**

In general, the results showed that the Farsi version of QSDI (eight factors and 39 items) had acceptable and applicable indices and it can be used as a valid tool in different fields for higher education students of medical sciences.

**Supplementary Information:**

The online version contains supplementary material available at 10.1186/s12909-021-02932-0.

## Background

Throughout the course of study, students need educational advices to actualize their potentials and avoid educational problems [1]. The student- supervisors relation is one of the key aspects of higher education programs and research courses in particular [2]. Supervisors might undertake different roles such as a trainer, educator, friend, colleague, role model, and mentor. These roles are fulfilled through trust and mutual relationship between the student and supervisors [3]. An efficient relationship between the student and supervisors leads to a higher educational performance and more efficient learning process [4]. Positive outcomes are expected out of such relationship such as a higher self-confidence, learning motivation, and improvement of professional skills in students, positive learning experience [5], less fear and anxiety in students, lower risk of educational failure, and better provision of support to the student [4].

One of the key responsibilities of higher education students that requires interaction with supervisor is dissertation writing [6]. Composing dissertation is the last stage in MSc and PhD programs that should be conducted under supervision of an advisor and a supervisor [7]. Success of research projects highly depends on the interaction between the student and supervisors [8]. Therefore, advising professor plays a key role as the guide for the students throughout the program. In the case of an efficient and positive relationship between supervisors and student, the supervisors’ role can be fulfilled in the best way possible [9].

A positive relationship between the supervisors and students is featured with respect, trust, and a low level of interpersonal conflicts [10]. Therefore, ability to create an efficient relationship is one of the dominant characteristics of supervisors [11]. Failure to form a decent relationship between supervisors and student leads to problems for the student, instructors, and higher education system in return [3]. Therefore, a proper interaction between the supervisors and student has a notable effect on the quality of dissertation and students’ satisfaction [12]. Among the elements of quality of dissertation, some believe that the student-supervisors interaction is one of the most important [3]. This relation is one of the most important factors in the quality of higher education programs [13]. At present, the questionnaires that measure student- supervisors interaction in Iran are researcher-made questionnaires that are designed based on library studies and are not used as a standard questionnaire. Therefore, a standard questionnaire is necessary for conducting related studies in Iran.

There are several tools to examine the interactions between supervisor and students [14–16]. The questionnaire on supervisor-doctoral student interaction (QSDI) by Mainhard is one of these tools. The tool has 41 items designed based on Likert’s five-point scale with eight aspects viz. leadership, helping/friendly, understanding, responsibility/freedom, uncertain, dissatisfied, admonishing, and stricture [15]. A short examination of the tool revealed that it perfectly examines the student- supervisors interaction in different fields. Given the absence of a reliable tool for this purpose in Iran, the present study is an attempt to validate and assess psychometric properties of QSDI for MD and higher education students in Iran.

## Methods

### Design

The study was carried out as a methodological and validation work for cultural validation and psychometric properties assessment of QSDI among MD and higher education students in medical sciences from December 2019 to March 2020.

### Participants

The study population was MD, MSc and PhD graduates of medical sciences universities over the past 2 years. The participants were selected through convenience sampling and the questionnaires were filled out in presence of researchers or sent to the participants as online copy (via e-mail and social networks).

The sample size for face validity stage consisted of 20 MD, MSc and PhD graduates and in content validity stage consisted of 16 professors. For construct (218 and 410) validity and reliability stage, 410 MD, PhD, and MSc. students of medical sciences were selected. For perform EFA, at first 218 students were selected by convenience sampling methods and then, the samples were increased to 410 students and CFA was performed.

Graduated MD and MSc students over the past 2 years and desire to participate were the inclusion criteria and failure to fill more than 80% of the content was the exclusion criterion.

### Questionnaire on supervisor-doctoral student interaction (QSDI)

In addition to demographics form, QSDI was used as the tool in the study. The QSDI was introduced by Mainhard et al. in the Netherlands with 41 items. The items are designed based on Likert’s five-point scale (never, rarely, sometimes, mostly, and always) and score of each item ranges from 1 to 5. In the case of direct items, the score “1” is assigned to “never” and “5” is assigned to always. Some of the items are inversely scored (items No. 2, 3, 4, 5, 7, 10, 11, 14, 15, 16, 18, 20, 21, 23, 26, 29, 32, 35, 37, 39, and 41). The tool has eight subscales and Cronbach’s alpha of each item is listed in Table 1 [15].

**Table 1:** QSDI, the sub-scales and Cronbach’s alpha

Dimension	Number of items	Cronbach’s alpha
Leadership (DC)	6	.86
Helping/friendly (CD)	6	.87
Uncertain (SO)	6	0.79
Dissatisfied (OS)	6	.71
Strict (DO)	5	.70
Understanding (CS)	4	.75
Student Responsibility/Freedom (SC)	4	.83
Admonishing (OD)	4	.71

### Cultural validity

Translation and cultural validation of the tool was based on Wild’s (2005) method [17] so that two Farsi translations of the tool were prepared by two native Farsi speakers and the translations were examined by the research team members. The two translations were merged and one copy was developed. Two other translators translated the Farsi version into English separately. The two English copies were examined, which is one of the main steps of cultural adaptation process. The translations were compared to the original version to spot difference and ensure conceptual similarity to the original copy. Eventually, the final copy was sent to the developer of the tool for confirmation.

To check cognitive similarity, the final copy was provided to 20 MD and MSc graduates in medical sciences to examine their perception, interpretation, and understanding of the translated copy. The tool was revised based on the results of cognitive information to ensure cultural comparability. Then, grammatical or spelling errors were checked and the final scale was prepared.

### Analysis

#### Descriptive analysis

Demographic variables of the research units were examined using relative frequency and content, mean and standard deviations of the mode.

#### Face validity

To check face validity, the scale was provided to another 20 PhD and MSc. graduates in medical sciences and in face-to-face interviews they were asked to highlight any vague item and word or ambiguity or wrong perception in the text.

#### Content validity

As to content validity, the tool was provided to 16 researchers, members of faculty boards, and experts of this subject from different disciplines. Their feedbacks were used to revise the scale and through this, content validity was ensured qualitatively. To determine content validity through quantitative method, content validity index (CVI) was obtained based on Walts and Bassel index [18] for each item (Table 2).

**Table 2:** The ratio and index of content validity Skewness and Kurtosis of the tool items

No	Items	CVR^a^	CVI^b^	Skew^c^		Kurt^d^	
	My supervisor...				cr		cr
1	always cooperates, if I want something	.67	.92	.13	1.05	−.26	−1.1
2	humiliates me	.83	.92	.21	1.74	−.75	−3.1
3	acts unconvincingly regarding my initiatives	.83	.83	−.35	−2.86	−.45	−1.86
4	is quick to criticize me	.83	.92	−.07	−.54	−.66	−2.7
5	is unclear during our conversations	.67	.92	−.32	−2.59	−.27	− 1.1
6	trusts me	.83	.83	−.57	−4.68	.06	.26
7	disbelieves me	.50	.92	−1.2	−10.2	1.46	6.1
8	helps me	.67	.92	−.02	−.2	−.61	−2.5
9	gives thorough feedback on my work	.83	.83	.02	.13	−.6	−2.48
10	has a bad temper during our discussions	.67	.92	−.62	−5.14	−.34	−1.38
11	is dissatisfied about my progress	.83	.92	−1.42	−11.7	1.5	6.3
12	follows my proposals	.50	.83	.15	1.2	−.78	−3.2
13	anticipates possible misunderstandings between us	.67	.92	.55	4.53	−.27	−1.12
14	thinks I know nothing	.83	.92	−.78	−6.4	−.34	−1.4
15	is impatient towards me	.67	.83	−.67	−5.56	−.3	−1.26
16	is critical of my work	.67	.75	.05	.37	.05	.2
17	listens to me	.83	.92	.14	1.1	−.99	−4.1
18	creates an atmosphere of ambiguity during our meeting	.67	.83	−.32	−2.7	−.65	−2.7
19	is strict when evaluating my progress	.83	.75	.28	2.3	−.07	−.3
20	demands a lot from me	.67	.83	−.05	−.39	−.64	−2.66
21	acts confidently when discussing my papers	.67	.92	.05	.44	−.24	−1.08
22	says that I am unskilled	.50	.83	.21	1.3	−.26	−1.1
23	always explains comprehensibly when I ask something	.50	.92	−.24	−2.02	−.09	−.36
24	gives me clear guidance	.67	.83	−.15	−1.2	.12	.49
25	thinks that I am dishonest	.50	.92	.18	1.25	−.15	−2.1
26	supports me	.67	.92	.27	2.22	−.71	−2.92
27	gives me a lot of advice	.67	.83	.36	2.95	−.29	−1.2
28	is indecisive about my initiatives	.83	.92	−.48	−3.99	.04	.18
29	acts professionally during our meetings	.83	.92	−.18	−1.5	−.08	−.32
30	reacts enthusiastically about my initiatives	.83	.83	.44	3.67	−.95	−3.91
31	acts irritable with me	.83	.92	.36	3	−.7	−2.88
32	is someone I can rely on	.67	.83	−.1	−.86	−.5	−2.04
33	pays attention, if I have something to say	.67	.75	.11	.92	−.75	−3.1
34	is uncertain during our meetings	.50	.83	−.3	−2.48	−.23	−.95
35	allows me to make my own decisions	.50	.92	.2	1.68	−1.15	−4.77
36	believes that I am untrustworthy	.67	.83	−.65	−5.38	0.29	1.2
37	shares my sense of humor	.83	.83	.06	.47	−.83	−3.44
38	is timid in our discussions	.67	.75	.19	1.6	−.52	−2.14
39	let’s me choose my own direction	.67	.83	−.82	−6.8	.56	2.3
40	is easily impressed by me	.50	.92	.04	.36	−.89	−3.67
41	immediately corrects me if I do something wrong	.83	.83	.36	2.94	.31	1.3
	Multivariate (Mardia test)					834.27	149.36

^a^Content Validity Ratio, ^b^Content Validity Index, ^c^Skewness is a measure of symmetry, or more precisely, the lack of symmetry, ^d^Kurtosis is a measure of whether the data are heavy-tailed or light-tailed relative to a normal distribution,

#### Construct validity

To check construct validity, exploratory factor analysis (EFA) and confirmatory factor analysis (CFA) were used.

In each stage of EFA and CFA, normal distribution of the data was checked using multivariate test.

In each stage of EFA and CFA, normal distribution of the data was checked using multivariate test. Because CFA with the maximum likelihood method is based on the assumption of normal distribution of data, the normal distribution of one and also multivariate data was examined. Skewness value for each statement varied from − 1.42 to 0.55 and it was at (− 2, 2) range. This means that, the statements are normal in terms of skewness with symmetric distribution [19]. Moreover, Kurtosis ranged from − 1.15 to 1.5 (Table 2). To discuss multivariate normality (Mardia test), if the critical ratio (cr) (Mardia test) for kurtosis is less than seven, the multivariate normality is rejected. In this study, the critical ratio (cr) (Mardia test) was 149.357, which was greater than seven and the normal distribution was supported [19, 20].

In addition, given factor load of each item (for t-value> 1.96, *p*-value = 95%; for t-value> 2.576, *p*-value = %99; and for t-value > 3.29, *p*-value = %999), to examine goodness of fit of the model, maximum likelihood method was used. To check reliability of the scores, internal consistency method was used through computing Cronbach’s alpha for each item and the whole tool.

## Results

### Descriptive results

In the case of EFA, totally, 57.3% of the participants were female and 42.7% were male. The mean age of 218 participants was 30.4 ± 6 with minimum and maximum ages equal to 24 and 51 years respectively. The mean time duration of preparing the dissertation from the approval of proposal to approval of the final dissertation was 16.2 ± 8.1 months (min = 6 months; max = 48 months).

In the case of CFA, totally, 56.1% of the participants were female and 43.9% were male. The mean age of 410 participants was 30.22 ± 5.92 with minimum and maximum ages equal to 24 and 51 years respectively. The mean time duration of preparing the dissertation from the approval of proposal to approval of the final dissertation was 16.14 ± 7.9 months (min = 6 months; max = 48 months). See Table 3 for the rest of demographics.

**Table 3:** Demographic characters of participants in study

Variables		EFA	CFA
		N (%)	N (%)
Gender	Male	93 (42.7)	180 (43.9)
	Female	125 (57.3)	230 (56.1)
Residence Location	Family house	129 (59.2)	261 (63.7)
	Student dormitory	89 (40.8)	149 (36.3)
Course grade	MD	114 (52.3)	217 (52.9)
	MSc	79 (36.2)	152 (37.1)
	PhD	25 (11.5)	41 (10)
Field of Study	Medicine	104 (47.7)	204 (49.8)
	Pharmacy	12 (5.5)	18 (4.4)
	Dentistry	17 (7.8)	29 (7.1)
	Nursing & Midwifery	63 (28.9)	117 (28.5)
	Health	22 (10.1)	42 (10.2)
Supervisor gender	Male	53 (24.3)	84 (69.3)
	Female	165 (75.7)	126 (60.7)
Supervisor degree	Lecturer	13 (6)	21 (5.1)
	Assistant Professor	101 (46.3)	191 (46.6)
	Associate Professor	77 (35.3)	159 (38.8)
	Professor	27 (12.4)	39 (9.5)

### Construct validity results

Before EFA, correlation of coefficient between the items was checked. Kaiser-Meyer-Olkin (KMO) test was obtained equal to 0.829 and Bartlett’s test of sphericity (χ2 = 10,932.91, sig = 0.0001) based on KMO test (0.7<) supported adequacy of correlation in the data. Moreover, *p*-value of Bartlett’s test was less than 0.05. Therefore, the required conditions for KMO were met. Principle components (PC) and Varimax Rotation were used for extracting the factors.

Communality value of all items was higher than 0.5. Therefore, none of the items were deleted in this stage and the rest of analyses were done on the 41 items.

To determine the number of factors, those with eigenvalue percentage higher than 1 were selected. The primary findings showed that eight factors can be selected for analysis. Additional file 1: Annexed Table 1. lists the factors extracted along with the eigenvalue, share of each factor in the variance of 41 items, and accumulated variance by each nine items.

**Fig. 1:**
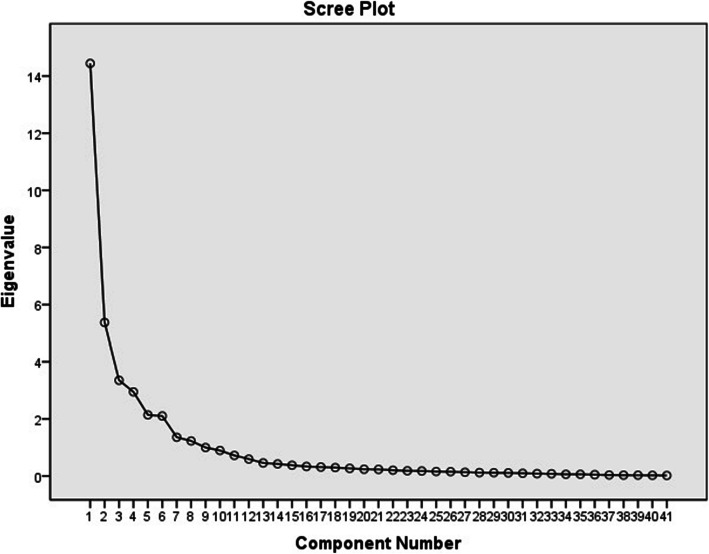
Scree plot of the extracted elements of the questionnaire

Totally, 80.34% of the variance of 41 items can be attributed to the eight items with eigenvalue> 1. The scree plot showed that the nine factors can be used for final analysis (Additional file 1: Annexed Table 2 and Fig. 1).

**Fig. 2:**
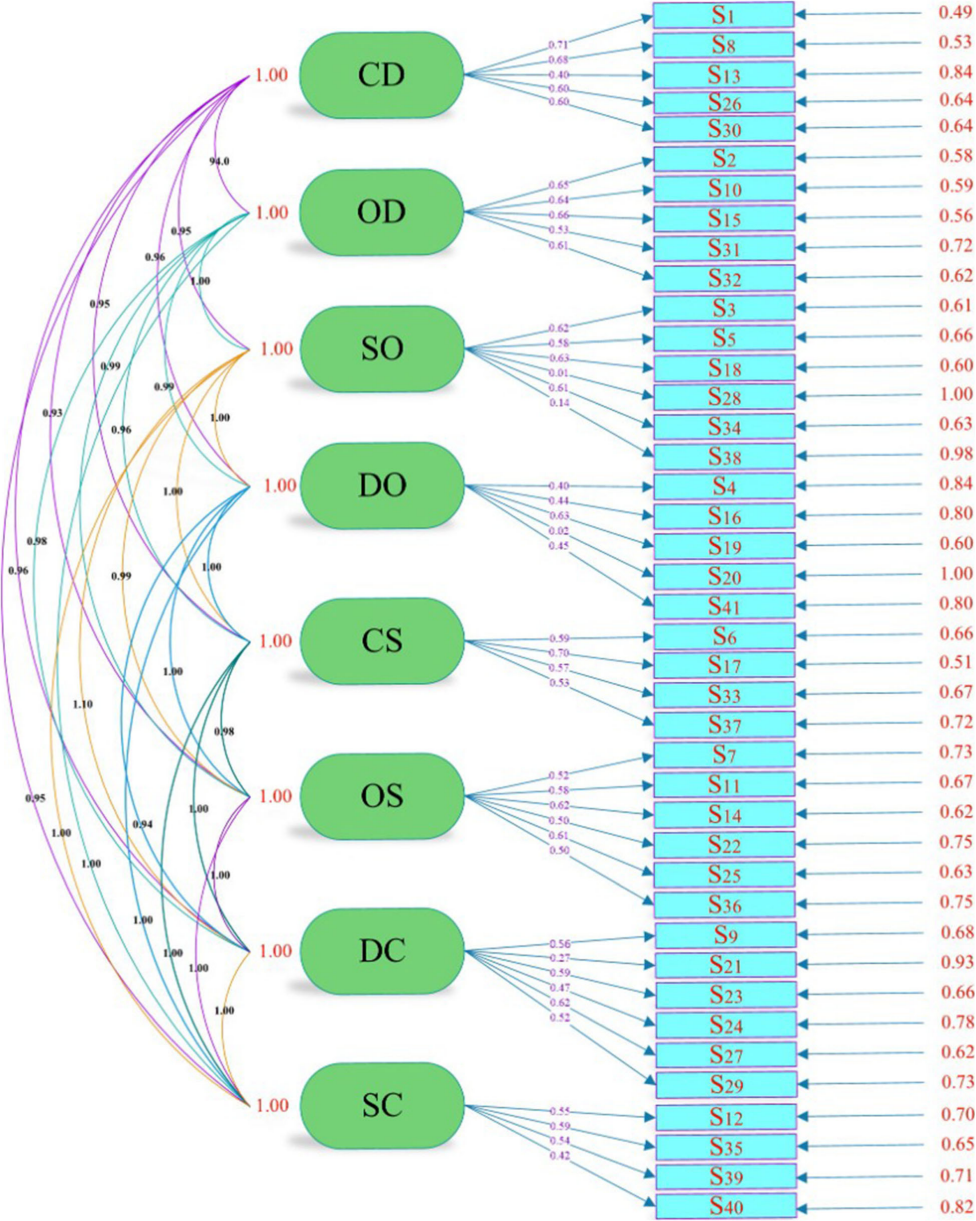
Eight factor model of CVI in QSDI in Iranian students- Standard

The CFA was conducted on eight factors and 41 items. Table 4. and Fig. 2. illustrate the results of CFA in standard coefficient modes.

**Table 4:** Results of CFA and reliability and consistency coefficients of sub-scale of measurement of QSDI

Factors		Items	Mead(sd)	T(cr)^a^	λ^b^	Cronbach’s alpha	
		My supervisor				Items	Factors
CD	1	always cooperates, if I want something	4.32(.92)	15.83	.71	.87	.893
	8	helps me	4.3(.88)	14.94	.68	.83	
	13	anticipates possible misunderstandings between us	3.94 (1.22)	8.06	.40	.67	
	26	supports me	4.45(.79)	12.7	.60	.59	
	30	reacts enthusiastically about my initiatives	4.28(.93)	12.7	.60	.81	
OD	2	humiliates me	4.72(.68)	14.14	.65	.93	.936
	10	has a bad temper during our discussions	4.65(.72)	13.92	.64	.91	
	15	is impatient towards me	4.65(.74)	14.59	.66	.92	
	31	acts irritable with me	4.56(.8)	11.13	.53	.93	
	32	is someone I can rely on	4.68(.74)	13.47	.62	.92	
SO	3	acts unconvincingly regarding my initiatives	3.74 (1.35)	13.57	.62	.95	.959
	5	is unclear during our conversations	3.69 (1.35)	12.42	.58	.95	
	18	creates an atmosphere of ambiguity during our meeting	3.69 (1.34)	13.78	.63	.95	
	28	is indecisive about my initiatives	3.29 (1.39)	0.13	.01	.97	
	34	is uncertain during our meetings	3.7 (1.32)	13.19	.61	.94	
	38	is timid in our discussions	3.59 (1.39)	2.7	.14	.96	
DO	4	is quick to criticize me	3.91 (1.06)	8.15	.40	.84	.894
	16	is critical of my work	3.99(.98)	9.03	.44	.91	
	19	is strict when evaluating my progress	4.05 (1.05)	13.00	.63	.87	
	20	demands a lot from me	3.73 (1.13)	−0.41	−.02	.87	
	41	immediately corrects me if I do something wrong	3.85 (1.09)	9.12	.45	.86	
CS	6	trusts me	4.35(.94)	12.51	.59	.91	.932
	17	listens to me	4.32(.97)	15.53	.70	.9	
	33	pays attention, if I have something to say	4.22 (1.03)	12.17	.57	.91	
	37	shares my sense of humor	4.08 (1.11)	11.11	.53	.93	
OS	7	disbelieves me	4.32 (1.05)	11.01	.52	.97	.97
	11	is dissatisfied about my progress	4.32 (1.04)	12.47	.58	.96	
	14	thinks I know nothing	4.3 (1.02)	13.53	.62	.96	
	22	says that I am unskilled	4.35 (1.02)	10.67	.50	.968	
	25	thinks that I am dishonest	4.33 (1.02)	13.28	.61	.964	
	36	believes that I am untrustworthy	4.34 (1.02)	10.47	.50	.964	
DC	9	gives thorough feedback on my work	4.18 (1.04)	12.03	.56	.91	.93
	21	acts confidently when discussing my papers	3.78 (1.26)	5.5	.27	.94	
	23	always explains comprehensibly when I ask something	4.27 (1.02)	12.59	.59	.91	
	24	gives me clear guidance	4.12 (1.06)	9.82	.47	.92	
	27	gives me a lot of advice	4.25(.98)	13.45	.62	.91	
	29	acts professionally during our meetings	4.1491.02)	10.95	.52	.91	
SC	12	follows my proposals	4.09(.94)	11.7	.55	.89	.932
	35	allows me to make my own decisions	4.22(.99)	12.63	.59	.87	
	39	let’s me choose my own direction	4.08(.999)	11.36	.54	.86	
	40	is easily impressed by me	4.04 (1.02)	8.81	.42	.89	
		QSDI (39 items)					.943

^a^The calculated values of t for all factor loads of the first and second order are greater than 1.96 and are therefore significant at the 95% confidence level, ^b^The specific value, which is denoted by the Landa coefficient and the statistical symbol λ, is calculated from the sum of the factors of the factor loads related to all the variables of that factor

Since t-values of items no.20 and 28 were less than 1.96, these two items were omitted (Table 4). Table 5 lists goodness of fit indices of CFA, which supports fitness of the model with the obtained data.

**Table 5:** Fit indicators confirmatory factor analysis persian version of QSDI

Fit indicators	Criterion	Level
χ^2^/DF	3≥	1.76
CFI	9.<	.98
NNFI/TLI	9.<	.98
GFI	9.<	.91
RMSEA	05.>	.043
R-square		.99
SRMR	05.>	.045
df		751
Chi-Square		1323.55
P_value_		1.00

Table 5 lists the final results of EFA and CFA, which were completed with eight factors and 39 items. Pearson correlation test indicated a positive and significant correlation (*p* < 0.001) between QSDI subscales and the whole scale (Table 6).

**Table 6:** Correlation coefficients of scale factors together and with the whole scale

		DC	CD	DO	SO	CS	OS	SC	OD	QSDI
DC	R	1								
	Sig.									
CD	R	.686^**^	1							
	Sig.	.03								
DO	R	.632^**^	.66^**^	1						
	Sig.	.000	.000							
SO	R	.721^**^	.693^**^	.673^**^	1					
	Sig.	.000	.000	.000						
CS	R	.573^**^	.611^**^	.567^**^	.656^**^	1				
	Sig.	.000	.000	.000	.000					
OS	R	.685^**^	.982^**^	.661^**^	.688^**^	.638^**^	1			
	Sig.	.000	.000	.000	.000	.000				
SC	R	.647^**^	.642^**^	.653^**^	.735^**^	.625^**^	.666^**^	1		
	Sig.	.000	.000	.000	.000	.000	.000			
OD	R	.683^**^	.707^**^	.707^**^	.733	.636	.694^**^	.692	1	
	Sig.	.000	.000	.000	.000	.000	.000	.772		
QSDI	R	.846^**^	.748^**^	.728^**^	.884^**^	.776^**^	.844^**^	.833^**^	.872^**^	1
	Sig.	.000	.000	.000	.000	.000	.000	.000	.000	

** *P* < .01

### Internal and external validity results

To check internal reliability, Cronbach’s alpha was obtained for the scale equal to 0.943. In the case of subscales, Cronbach’s alpha was between 0.89 and 0.97 so that the subscales have the required reliability (Table 6). All of reliability coefficients of the factors and the questionnaire itself were at the desired level.

As listed in Table 6, there is a positive and significant correlation between QSDI and all of factors (subscales) and also between the factors. In general, at the end of the two stages (EFA and CFA) and after removing items No. 20 and 28 (in the CFA method), eight factors and 39 items were extracted as described in the Table 6.

## Discussion

Cultural validation and psychometrics of Farsi version of QSDI for PhD and MSc students in medical science universities in Iran were examined. After obtaining content validity through qualitative method, content validity was obtained through quantitative method as a supplementary measure. To this end, CVR and CVI were obtained and content validity of the tool was supported. This indicated good cultural validity of the scale for Iranian society.

The EFA and CFA were used for construct validity. Reliability and internal validity of the scale were checked using Cronbach’s alpha and the correlation coefficients among the factors respectively. The EFA results confirmed the scale with eight factors and 41 items, the original tool has eight factors and 41 items [15]. To measure construct validity of student-supervisor relationship scale, Ali et al. (2016) used EFA [21]. Castello et al. used EFA to check construct validity of the tool. Based on the EFA results, some of the subscales were removed [22, 23]. To elaborate on the findings, in addition to the effect of culture and educational structure on respondents’ answers, it is notable that the developer of the scale has not examined construct validity of the tool. The present study, however, used EFA as a key measure of construct validity of the scale [24, 25].

The results of CFA confirmed the scale with eight factors and 39 items (CFI > 0.9; NNFI, GFI > 0.8, and RMSEA = 0.043). The original scale has eight factors and 41 items [15]. Removal of two items from the original scale based on EFA and CFA results can be explained by the different research environment, Iranian culture, and number of participants.

The results showed that the 39-item scale obtained through factor analysis two items eliminated in CFA) has a positive and significant correlation with its eight subscales. In addition, the results supported internal stability of the scale and Cronbach’s alpha for the 39-item scale was equal to 0.96. Cronbach’s alphas of leadership, helping/friendly, understanding, responsibility/freedom, uncertain, dissatisfied, admonishing, and strict were equal to 0.93, 0.893, 0.932, 0.91, 0.959, 0.97, 0.936, and 0.894 respectively; these figures in Minhard’s et al. study were 0.71, 0.73, 0.75, 0.7, 0.71, 0.79, 0.87, and 0.86 respectively [15]. Clearly, the eight factors in the present study are consistent with Mainhard’s et al., which can be explained based on cultural differences, participants’ difference, and number of participants.

Minhard et al. reported that there was an inverse correlation between SO, OS, and OD and Dc, CD, CS, and SC. In addition, the correlation between DO and CS, SC, and SO was direct [15]. The present study, however, showed that the positive and significant correlation between all factors.

Consistent with the results of the initial study [15], the model has eight axes and each of which can have a significant impact on the model. In Minhard et al., DC-leadership, CD-helping / friendly, CS-understanding, and SC-student responsibility / freedom had a higher degree of proximity than the other four axes. In terms of influences, the OD-admonishing, DO-strict, DC-leadership and CD-helping / friendly axes had a stronger influence than the other four axes.

In the present study, due to the type of questions and the scoring method, some questions are reversely scored. No such cases were seen in our analysis of the results, because the data analysis method was different. To confirm the validity of the structure from EFA and CFA is used.

### Limitation of study

One of the limitations of the study was the lengthy process of administration of the questionnaires due to the limitations of COVID-19 as the universities were closed. The questionnaires were distributed as had copy or as electronic form. This study was conducted on academic participants and, therefore, their international interactions can greatly reduce the existing cultural differences, which was one of the limitations of the study. Due to the time limitations and available sample during COVID-19 pandemic, EFA and CFA were performed on one the same sample group. However, CFA was done to complete the study. Therefore, CFA of this questionnaire with larger and different samples is recommended.

## Conclusion

In conclusion, the Farsi version of QSDI with eight factors and 39 factors is a valid and reliable tool for Iranian society and it can be used for PhD and MSc students in medical sciences in Iran.

## Supplementary Information

**Additional file 1: Annexed Table 1.** Matrix of factor loads of the questionnaire questions on components after rotation. **Annexed Table 2.** Percentage of variance and eigenvalues of different factors

## Data Availability

The datasets used in the study are available from the corresponding author on reasonable request.

## Abbreviations

MD: Medical Doctor
MSc: Master of Sciences
Ph.D.: Philosophiae Doctor
QSDI: Questionnaire on supervisor-doctoral student interaction
CVI: Content Validity Index
CVR: Content Validity Ratio
KMO: Kaiser Meyer Olkin
EFA: Exploratory Factor Analysis
CFA: Confirmatory Factor Analysis
TLI: Tucker-Lewis Index
NFI: Normed Fit Index
GFI: Goodness of Fit Index
RMSEA: Root Mean Square Error of Approximation
PC: Principal Components
SRMR: Standardized Root Mean Square Residual
KUMS: Kermanshah University of Medical Sciences
