# Supplementary material for: Assessment of psychometric properties of the questionnaire on supervisor-doctoral student interaction (QSDI) in Iran
Source: BMC Med Educ. 2021 Oct 14;21:531. doi: 10.1186/s12909-021-02932-0 (PMC8515155; doi:10.1186/s12909-021-02932-0)
Supplement: Supplementary file 1 — Additional file 1: Annexed Table 1. Matrix of factor loads of the questionnaire questions on components after rotation. Annexed Table 2. Percentage of variance and eigenvalues of different factors [file 12909_2021_2932_MOESM1_ESM.docx]

| **Annexed Table1. Matrix of factor loads of the questionnaire questions on components after rotation** | | | | | | | | |
| --- | --- | --- | --- | --- | --- | --- | --- | --- |
|  | Component | | | | | | | |
|  | 1 | 2 | 3 | 4 | 5 | 6 | 7 | 8 |
| s1 | .249 | .033 | .300 | .159 | .179 | .103 | .754 | .295 |
| s2 | .103 | -.015 | .154 | .823 | .107 | .059 | .172 | .120 |
| s3 | -.007 | .948 | .029 | -.019 | -.035 | .020 | .034 | -.007 |
| s4 | -.006 | -.039 | .079 | .131 | .901 | -.036 | .063 | .145 |
| s5 | .044 | .933 | .069 | -.023 | -.043 | .060 | .055 | .069 |
| s6 | .415 | .130 | .220 | .155 | .120 | .017 | .149 | .683 |
| s7 | .773 | -.043 | .259 | .113 | .061 | .064 | .227 | .234 |
| s8 | .334 | -.028 | .274 | .191 | .140 | .133 | .729 | .163 |
| s9 | .246 | .059 | .780 | .091 | .125 | .106 | .248 | .194 |
| s10 | .158 | -.008 | .159 | .882 | .123 | .074 | .090 | .125 |
| s11 | .840 | -.022 | .293 | .171 | .073 | .117 | .198 | .215 |
| s12 | .007 | .034 | .099 | .055 | .014 | .845 | .170 | .111 |
| s13 | .203 | -.175 | .163 | .112 | .083 | .119 | .638 | .373 |
| s14 | .827 | -.029 | .284 | .202 | .066 | .114 | .199 | .225 |
| s15 | .205 | .003 | .161 | .821 | .142 | .086 | .148 | .138 |
| s16 | .023 | -.144 | .221 | .179 | .590 | .069 | .131 | .212 |
| s17 | .347 | .146 | .233 | .185 | .132 | .030 | .203 | .734 |
| s18 | .022 | .933 | .047 | .025 | -.057 | .044 | -.002 | -.002 |
| s19 | .128 | .045 | .103 | .022 | .838 | .014 | .181 | .053 |
| s20 | .036 | -.125 | -.012 | .179 | .851 | -.075 | -.036 | -.004 |
| s21 | .164 | -.179 | .643 | .192 | .089 | .074 | .036 | .231 |
| s22 | .775 | -.011 | .332 | .185 | .044 | .085 | .198 | .197 |
| s23 | .299 | .098 | .840 | .137 | .031 | .051 | .095 | .026 |
| s24 | .233 | .020 | .823 | .113 | .056 | .047 | .142 | .120 |
| s25 | .750 | -.012 | .304 | .131 | .087 | .135 | .306 | .274 |
| s26 | .252 | .142 | .048 | .250 | .150 | .005 | .689 | -.077 |
| s27 | .246 | .119 | .841 | .119 | .096 | .084 | .205 | .125 |
| s28 | -.044 | .772 | -.010 | -.004 | -.074 | .031 | .008 | .169 |
| s29 | .274 | .057 | .808 | .196 | .077 | .140 | .084 | .082 |
| s30 | .406 | .023 | .232 | .159 | .070 | .143 | .688 | .242 |
| s31 | .108 | -.043 | .113 | .787 | .158 | .159 | .126 | .150 |
| s32 | .150 | .036 | .142 | .859 | .136 | .135 | .100 | .019 |
| s33 | .419 | .087 | .221 | .141 | .100 | .068 | .280 | .706 |
| s34 | -.006 | .960 | .049 | .011 | -.024 | .071 | -.009 | .000 |
| s35 | .221 | .151 | .121 | .129 | -.038 | .853 | -.001 | -.035 |
| s36 | .818 | -.005 | .283 | .155 | .080 | .180 | .195 | .181 |
| s37 | .329 | -.032 | .176 | .218 | .104 | .072 | .142 | .776 |
| s38 | -.041 | .885 | -.039 | -.011 | -.051 | -.028 | -.048 | -.075 |
| s39 | .092 | .044 | .096 | .149 | .022 | .888 | .073 | .049 |
| s40 | .119 | -.023 | .047 | .101 | -.050 | .855 | .038 | .014 |
| s41 | .105 | -.061 | .034 | .126 | .854 | -.003 | .102 | -.019 |
| Extraction Method: Principal Component Analysis.  Rotation Method: Varimax with Kaiser Normalization. | | | | | | | | |
| a. Rotation converged in 6 iterations. | | | | | | | | |

Annexed Table 1. Factors extracted after exploratory analysis

| Factors | Initial Eigenvalues | | | Extraction Sums of Squared Loadings | | | Extraction Sums of Rotated loads | | |
| --- | --- | --- | --- | --- | --- | --- | --- | --- | --- |
|  | Total | % of Variance | Cumulative % | Total | % of Variance | Cumulative % | Total | % of Variance | Cumulative % |
| 1 | 14.442 | 35.225 | 35.225 | 14.442 | 35.225 | 35.225 | 5.422 | 13.225 | 13.225 |
| 2 | 5.374 | 13.107 | 48.332 | 5.374 | 13.107 | 48.332 | 5.178 | 12.630 | 25.854 |
| 3 | 3.352 | 8.175 | 56.507 | 3.352 | 8.175 | 56.507 | 4.934 | 12.035 | 37.890 |
| 4 | 2.945 | 7.183 | 63.690 | 2.945 | 7.183 | 63.690 | 4.212 | 10.273 | 48.162 |
| 5 | 2.139 | 5.216 | 68.907 | 2.139 | 5.216 | 68.907 | 3.636 | 8.868 | 57.031 |
| 6 | 2.102 | 5.126 | 74.033 | 2.102 | 5.126 | 74.033 | 3.256 | 7.942 | 64.973 |
| 7 | 1.358 | 3.311 | 77.344 | 1.358 | 3.311 | 77.344 | 3.253 | 7.935 | 72.908 |
| 8 | 1.228 | 2.995 | 80.340 | 1.228 | 2.995 | 80.340 | 3.047 | 7.432 | 80.340 |
